# Complex symptoms of demyelination and nerve damage explained by nonlinear dynamical analysis of conductance-based models

**DOI:** 10.1186/1471-2202-12-S1-P292

**Published:** 2011-07-18

**Authors:** Jay S Coggan, Gabriel K Ocker, Steven A Prescott, Terrence J Sejnowski

**Affiliations:** 1 Computational Neurobiology Laboratory, The Salk Institute for Biological Sciences, La Jolla, CA, 92037, USA; 2 Department of Neurobiology and Pittsburgh Center for Pain Research, University of Pittsburgh, Pittsburgh, PA, USA; 3 Howard Hughes Medical Institute, USA

## 

Multiple sclerosis and other demyelination diseases are associated with positive (gain of function), negative (loss of function), and paroxysmal (sudden and short duration) symptoms. These symptoms occur in a remarkable diversity of modalities including prolonged or, in the case of paroxysmal symptoms, brief episodes of pain, paresthesia (numbness), dysarthria (difficulty speaking), weakness, muscle spasms, etc. [1]. Using computational modeling, we have recently established the mechanisms underlying four distinct phases of action potential (AP) firing associated with demyelination and secondary compensatory changes [2]. Transitions in axonal excitability caused by varying the ratio of *g_Na_* and *g_L_* indicate that a single mechanism controlling conductance balance is sufficient to explain normal spiking, failed conduction, afterdischarge (AD) and spontaneous activity, and can thus account for the full range of negative, paroxysmal and tonic positive symptoms, respectively. Positive symptoms are thought to arise from ectopic spike discharge, but the biophysical mechanisms responsible for the abrupt onset and self limiting duration of paroxysmal AD are poorly understood and were thus a particular area of focus.

We used two types of complementary models. The first was a Hodgkin-Huxley (HH), multi-compartment model of a focally demyelinated axon created using the NEURON Simulator (http://www.neuron.yale.edu/neuron, [3]) based on a geometrically simplified model to first understand the behaviors of electrical conductances. The second was a modified Morris-Lecar, single compartment model [4] that has been described in detail [5]. We explored this model with phase-plane and bifurcation analysis using XPPAUT in order to rigorously characterize the dynamical mechanisms underlying phenomena identified in the HH model.

In both models, the paroxysmal AD can only occur when the system is bi-stable and initiation occurs when a perturbation abruptly switches the system between attractor states. Termination of paroxysms occurs when internal dynamics shift the basins of attraction until the system falls back to its original stable state. Temporal summation and refractoriness can also be simply explained within this framework. Disruption of the oligodendrocytes that form the myelin sheaths may lead to abnormal electrolyte homeostasis in the vicinity of the denuded axon. Our models indicate that the pathological accumulation of Na^+^ or K^+^ can influence the duration of paroxysmal firing by acting as an additional, ultra-slow feedback mechanism in the dynamical system. As such, a naked patch of previously myelinated axonal membrane becomes capable of competing with the same neuron’s soma for the initiation of burst firing patterns and interferes with normal firing patterns. Although not biologically detailed, our modeling nonetheless provides significant insight into how biologically realistic mechanisms can interact to produce neuronal discharges as well as other abnormal firing characteristics in the course of demyelination.

This work was supported by the Howard Hughes Medical Institute (TJS), NIH 5R01MH079076 (TJS) and start-up funds from the University of Pittsburgh (SAP). SAP is also a Rita Allen Foundation Scholar in Pain
